# Suicidal ideation among Lebanese adults: scale validation and correlates

**DOI:** 10.1186/s12888-021-03111-7

**Published:** 2021-02-16

**Authors:** Maha Zakhour, Chadia Haddad, Hala Sacre, Kassandra Fares, Marwan Akel, Sahar Obeid, Pascale Salameh, Souheil Hallit

**Affiliations:** 1 Faculty of Science, Lebanese University, Fanar, Lebanon; 2 INSERM, University Limoges, CH Esquirol, IRD, U1094 Tropical Neuroepidemiology, Institute of Epidemiology and Tropical Neurology, GEIST, Limoges, France; 3 INSPECT-LB: National Institute of Public Health, Clinical Epidemiology and Toxicology, Beirut, Lebanon; 4 Faculty of Arts and Sciences, Holy Spirit University of Kaslik (USEK), Jounieh, Lebanon; 5 School of Pharmacy, Lebanese International University, Beirut, Lebanon; 6 Research and Psychology Departments, Psychiatric Hospital of the Cross, Jall-Eddib, Lebanon; 7 Faculty of Pharmacy, Lebanese University, Hadat, Lebanon; 8 Faculty of Medicine, University of Nicosia, Nicosia, Cyprus; 9 Faculty of Medicine and Medical Sciences, Holy Spirit University of Kaslik (USEK), Jounieh, Lebanon

**Keywords:** Suicidal ideation, Depression, Anxiety, Alexithymia, Emotional intelligence

## Abstract

**Background:**

According to the World Health Organization (WHO) figures in 2015, the number of people attempting suicide worldwide per year exceeds 800,000 individuals. The majority of completed suicides (78%) occur in low- and middle-income countries. Therefore, this study aimed to validate the suicidal ideation subscale of the Columbia-Suicide Severity Rating Scale and evaluate risk factors (emotional intelligence, alexithymia, anxiety, depression, and stress) related to suicidal ideation among the Lebanese adult population.

**Methods:**

A structured cross-sectional survey was carried out between November 2017 and March 2018, enrolling a proportionate random sample of 789 community-dwelling participants from all the Lebanese regions. A correlation analysis between the C-SSRS and anxiety and depression assessed the convergent validity of the scale. An exploratory and a confirmatory factor analysis validated its construct. Cronbach’s alpha was used to assess internal consistency reliability. Multiple linear regression was performed using the suicidal ideation score as the dependent variable. All variables were included in the multivariable model.

**Results:**

The C-SSRS scale converged over a solution of one factor; the proportion of explained variance was 0.797. The Cronbach’s alpha value was good (0.797). The convergent validity was tested with depression and anxiety scales. The results showed a moderate positive correlation between the suicide ideation score and depression (rho = 0.507, *p* < 0.001) and anxiety (rho = 0.402, *p* < 0.001). The multivariable analysis showed that higher depression (Unstandardized Beta, B = 0.035, p < 0.001), higher anxiety (B = 0.015, *p* = 0.008), and higher alcohol dependence (B = 0.024, p < 0.001) were significantly linked to higher suicidal ideation score. However, higher self-esteem (B = -0.041, *p* = 0.006) was significantly associated with lower suicidal ideation scores.

**Conclusion:**

Our findings suggest that the Arabic version of the C-SSRS subscale could serve as an appropriate assessment tool for suicidal ideation. This paper also gave insights into factors correlated with higher suicidal ideation scores, such as depression, anxiety, and alcohol dependence. Further studies are necessary to confirm our findings and implement suicide prevention programs.

## Background

Suicide is a considerable health problem worldwide; it is one of the top fifteen causes of death across the global population and the second leading cause in young adults [1, 2]. According to the World Health Organization (WHO) figures in 2015, the number of people attempting suicide worldwide per year exceeds 800,000 individuals [3]. In the United States, suicide is the 10th leading cause of death for all ages; the suicidal rate increased by 35% from 1999 through 2018, for both males and females [4]. In 2017, 1.4% of global deaths were from suicide [3]. The majority of completed suicides (78%) occur in low- and middle-income countries.

Studies have identified several acute and chronic risk factors for suicide, most notably suicidal ideation and attempts and other indicators of mental health, substance use, and psychosocial resources. Suicide thoughts and attempts are predictors of suicide deaths [3, 5, 6]. Suicidal ideation refers to any intention to die, kill self, or plan to end life [7, 8]. Women are 3 to 4 times more likely to attempt suicide, while men are more likely to commit suicide [9]. Overall, for every 20 suicidal attempts, one complete suicide is realized [10]. The acute state, known as the suicide crisis syndrome, is identified as the short term suicidal risk, while chronic risk factors are referred to as long term suicidal risk [11]. Chronic risk factors include mental illness, suicidal ideation, previous suicide attempts, severe hopelessness, a history of childhood maltreatment, insecure attachment, chronic substance use, impulsivity, lack of social support, and perfectionism [11, 12]. Acute risk factors for suicide include sleep disturbances, loss of concentration and interest, severe anxiety, irritability, anhedonia, and social isolation [13]. Depression is the most prevalent psychiatric disorder in suicide attempts [14, 15]. It plays a central role in the transition from suicidal ideations to suicide attempts [16]. Depressed persons lack energy, motivation, and initiative; they also have persistent fatigue and sadness. Anxiety was not shown to be directly related to suicidal attempts: anxious individuals may refrain from having suicidal thoughts because they fear harm [17]. However, the avoidance behaviors characterizing anxiety disorders lead to functional disability that may increase vulnerability to suicide [18]. Also, social anxiety was seen as an indicator of suicidal ideation, as addressing the feeling of loneliness and low perception of social support may increase suicidal ideation and attempts [19]. Panic attacks were also identified as a risk factor for attempted suicide among depressed persons with or without suicidal ideation [20].

Researchers have been accumulating evidence that some stressful conditions may increase the risk for suicidal ideation and attempts [21–23], such as an overloading persisting situation, constant stress, and negative life experiences or incidents, leading to mental health disorders and suicidal ideation [23]. Low self-esteem was also linked to increased suicidal tendencies and tentative [24–26]. A constant negative view of oneself may include seeing oneself as worthless, the future as hopeless, and life as not worth living [27]. The consumption of alcoholic beverages has also been associated with suicidal thoughts and attempts [28]. Drinking alcohol by itself cannot lead to suicide; however, the consequence of alcohol addiction could include destructive behaviors such as losing a job, domestic and work problems, violent or criminal acts, and social withdrawal [29]. These factors can lead to depression, and subsequently, suicidal thoughts and actions [29]. Alexithymia, described as the inability to identify emotions or express them [30], has also been linked to suicide ideation [31]. People with alexithymia may develop emotional dysregulation and confusing information, leading to feelings of helplessness [32]. Individuals with poor emotional clarity have difficulty regulating their emotions; they use maladaptive coping mechanisms, such as social isolation, which contribute to suicide ideation and behavior because of limited ways of coping with the feelings they have [31]. Oppositely, the role of emotional intelligence (EI) in promoting positive emotions and well-being is crucial [33]. People with high EI people are likely to make and keep tight connections, express emotions and feelings, and improve their subjective well-being [34]. Thus, EI could be a protective factor against suicide [35].

In Lebanon, suicide rates have increased during the last years. Police data indicate that 100 suicides were recorded in the first 5 months of this year, compared to 147 suicides in all of 2017 and 200 suicides in all of 2018 [36]. Indeed, mental health conditions are common in the Lebanese population that has high mental health problems, similar to European countries like France, Italy, Belgium, and others [37]. A study among 2857 Lebanese adults has found that the lifetime prevalence of mental disorders was 25.8%, with 10.5% of the studied sample having more than one [38]. The projecting lifetime risk was approximately 32.9%, meeting the criteria for one or more of the DSM-IV disorders [38]. Different stressful factors increase suicidal ideations among the Lebanese population, such as the unstable political situation resulting from a 20-year civil war [39], environmental problems (chronic power shortage, lack of clean water, and waste mismanagement) [39], and the economic hardship translating into lack of vacancies and increased unemployment due to the competition with the Syrian refugees [40]. A study suggested that one of the most important reasons for the rising suicide rates could be Lebanon’s economic decline that worsened almost 2 years ago [36]. Another research recently explored suicidal ideation among Lebanese adolescents and found that 28.9% of teenagers had suicidal thoughts [41]; previous studies had shown that the prevalence of suicidal ideation among Lebanese adults was 12% [42, 43]. In Lebanon, the risk factors for suicide ideation identified among adolescents [44] included gender (females more at risk), depression, sadness, loneliness, alcohol and drug abuse, bullying, and several other factors [45].

Assessing suicidal thoughts and behaviors is essential to complement clinical evaluation and prevent suicide [46]. Several instruments have been used for this purpose [47], among which the Columbia-Suicide Severity Rating Scale or C-SSRS, designed to measure the severity of suicide ideation and type of suicidal behavior [48]. It has been translated into 125 languages, including the Lebanese Arabic language. The initial version of the C-SSRS was assessed in three multisite studies with adolescents and adults: the tool demonstrated a high internal consistency (α = 0.73 to 0.95), a strong integration (r = 0.80), and divergence, in addition to high sensitivity and specificity for suicidal behavior compared with other suicidal and behavior scales [48].

In Lebanon, most studies focused on adolescents, with the C-SSRS showing adequate reliability (α =  0.966) and good convergent validity with psychological scales [41]. However, it is not known whether this tool is valid and reliable for assessing suicidal ideation among adults. Therefore, this study aimed to validate the suicidal ideation subscale of the Columbia-Suicide Severity Rating Scale and evaluate risk factors (emotional intelligence, alexithymia, anxiety, depression, and stress) related to suicidal ideation among the Lebanese adult population.

## Methods

### Sampling and data collection

A structured cross-sectional survey was carried out between November 2017 and March 2018, enrolling a proportionate random sample of 789 community-dwelling participants from all the Lebanese regions. Out of 1000 questionnaires distributed, 789 (78.9%) were completed and collected back; 211 (21.1%) of the contacted people declined to participate.

Lebanon is divided into five Governorates (Mohafazat), Mount Lebanon, Beirut, North, Beqaa, and South, in turn, divided into Districts (Caza) from which two villages were randomly selected from the list provided by the Lebanese Central Agency of Statistics.

In each village, the questionnaires were distributed to the randomly selected household. All individuals above 18 were eligible. A clinical psychologist assessed participants’ cognitive abilities, and those deemed unable to respond and understand the questions were excluded from the study. People who agreed to participate were asked to fill out the survey form via a face-to-face interview. Those who did not know how to read or write were offered assistance.

Participants were briefed on the aims and methods of the study before enrolling. They had the right to accept or refuse to participate. Those who agreed signed written informed consent and received no financial compensation for their participation. Their anonymity was guaranteed during the data collection process. The same methodology was used in previous papers [49–52].

### Minimal sample size calculation

According to Comrey and Lee, at least ten observations per item are necessary to carry out an exploratory factor analysis [53]. Therefore, a minimum sample of 50 patients was required in this study since the suicidal ideation part of the C-SSRS questionnaire comprises of five items.

### Procedure

A study-independent clinical psychologist interviewed the participants, using a paper-pen method to collect the resulting data. Questions were written and asked in Arabic, the native language in Lebanon.

### Translation procedure

All scales were forward and backward translated, except for the Hamilton depression rating scale and the Hamilton anxiety scale, already validated in Lebanon [54, 55]. One bilingual translator translated the scales from English into Arabic. Another translator performed the back-translation from Arabic into English. Discrepancies were resolved by consensus.

### Questionnaire

The first section assessed sociodemographic and other characteristics of the participants, such as age, gender, and level of education. It also gathered information about their monthly income, classified into no income, low (< 1000 US dollars), intermediate (between 1000 and 2000 US dollars), and high (> 2000 US dollars). The second part included the various scales used in this survey.

#### The Columbia-suicide severity rating scale (C-SSRS)

The Columbia Suicide Severity Rating Scale (C-SSRS) is a 10-item dichotomous scale developed by researchers from the universities of Columbia, Pennsylvania, and Pittsburgh to evaluate suicidal ideation (5 questions) and behavior (5 questions) [48]. This study used the five questions about suicidal ideation: “wish to be dead”, “suicidal thoughts”, “suicidal thoughts with a method”, “suicidal intent”, and “suicidal intent with a specific plan”. A score evaluating suicidal ideation in the past week was created by summing the responses to the five items, where a score of “0” indicates no suicidal ideation. An answer of yes to any of the five questions signs the presence of suicidal ideation [56], with higher scores indicating more suicidal ideation (α_Cronbach_ = 0.796). The possible range for the suicidal ideation subscale was 0–5.

#### The alcohol use disorders identification test (AUDIT)

The AUDIT is a 10-item scale that measures alcohol consumption, drinking behaviors, and problems related to alcohol in the past year [57]. The assessment period was done during the last year. Examples of items: “How often do you have a drink containing alcohol?”, “How many drinks containing alcohol do you have on a typical day when you are drinking?”. Items 1 to 8 are graded on a scale from 0 (never) to 4 (daily or almost daily) [57]. Questions 9 and 10 have only three possible responses and are scored 0, 2, and 4 [57]. The total AUDIT score was used and ranged from 0 to 40. Scores of eight or above indicate alcohol use disorder (α_Cronbach_ = 0.885) [57].

#### The Toronto alexithymia scale (TAS-20)

This 20-item tool evaluates alexithymia over the last month. Answers are rated on a 5-point Likert scale from 1 (strongly disagree) to 5 (strongly agree) [58]. Examples of items: “I am often confused about what emotion I am feeling”, “It is difficult for me to find the right words for my feelings”, “I prefer to analyze problems rather than just describe them”. The total TAS-20 score was used and ranged between 20 and 100, with higher scores indicating higher alexithymia [58] (α_Cronbach_ = 0.778).

#### The Rosenberg self-esteem scale (RSES)

The RSES consists of 10 items that measure self-esteem over the last month [59]. Examples of items: “On the whole, I am satisfied with myself”, “At times I think I am no good at all”, “I feel that I have a number of good qualities”. All answers are graded on a 4-point Likert scale from 1 (strongly disagree) to 4 (strongly agree). The total score was used and ranged from 10 to 40. Higher scores indicate greater self-esteem (α_Cronbach_ = 0.733).

#### Hamilton depression rating scale (HDRS)

The HDRS, validated in Lebanon, is used to assess depression experienced over the past week [54]. It consists of 21 items, but the scoring is based on the first 17 only. Of these 17, eight are rated on a 5-point scale from 0 (not present) to 4 (severe) [60], and nine from 0 to 2. The total score is calculated by summing the responses to the 17 questions. Examples of items: “depressed mood”, “feelings of guilt”, “suicide”. The total HDRS score was used and ranged from 0 to a maximum of 52 points. Higher scores indicate higher depression [60] (α_Cronbach_ = 0.890).

#### Hamilton anxiety scale (HAM-A)

This tool, previously validated in Lebanon, is used to evaluate anxiety over the past week [55]. Examples of items: “Anxious mood”, “Tension”, “Fears”. Each question is rated on a 5-point scale from 0 (not present) to 4 (severe), with a total score ranging between 0 and 56 [61]. Higher scores indicate higher anxiety (α_Cronbach_ = 0.898).

#### The perceived stress scale (PSS)

The PSS consists of 10 items measuring the perception of stress during the last month. Examples of items: “In the last month, how often have you been upset because of something that happened unexpectedly?”, “In the last month, how often have you felt that you were unable to control the important things in your life?”, “In the last month, how often have you felt nervous and stressed?”. Responses are graded on a scale from 0 (never) to 4 (very often) [62]. The total score was used and ranged from 0 to 40, with higher scores indicating higher perceived stress (α_Cronbach_ = 0.667).

#### Liebowitz social anxiety scale (LSAS)

The LSAS is a 24-item self-report scale that measures social anxiety disorder over the past week [63]. Questions are divided into two subcategories evaluating fear and avoidance. Examples of items: “Participating in a small group activity”, “Drinking with others”, “Acting, performing, or speaking in front of an audience”. Questions are rated on a scale from 0 (none for fear, or never for avoidance) to 3 (severe for fear and usually for avoidance). The total score calculated by summing the answers to the two subcategories varies between 0 and 144. A higher score indicates very severe social phobia. The Cronbach’s alpha values for the total score, avoidance, and fear scores were 0.954, 0.953, and 0.945, respectively.

#### The quick emotional intelligence self-assessment

This scale consists of 40 items divided into four subscales of ten questions: emotional awareness, emotional management, social-emotional awareness, and relationship management. Items are scored on a Likert scale from 0 (never) to 4 (always). Examples of questions: “My feelings are clear to me at any given moment”, “I accept responsibility for my reactions”, “I consider the impact of my decisions on other people”, “I am able to show affection”. The total score was used and calculated by summing all items of the four subscales and ranges from 0 to 160 stress (α_Cronbach_ = 0.958) [64].

### Statistical analysis

Data were analyzed using SPSS software version 25. A descriptive analysis was carried out using numbers and percentages for categorical variables and means and standard deviations for continuous measurements. Factor analysis and confirmatory factor analysis validated the construct of the C-SSRS scale. Factor analysis using the Principal Component Analysis (PCA) was performed via the “FACTOR” procedure. The Kaiser-Meyer-Olkin sampling adequacy measure and Bartlett’s sphericity test were appropriate. All factors retained had an Eigenvalue over 1. A promax rotation was performed because the extracted factors were correlated. This procedure was followed by a Confirmatory Factor Analysis (CFA), which examined the fit of the factor model of the suicidal ideation score. The following goodness-of-fit indicators were reported: the chi-square to df ratio (χ2/df), the Root Mean Square Error of Approximation (RMSEA), the Goodness-of-fit statistic (GFI), and the adjusted goodness-of-fit statistic (AGFI). The χ2/df having a low sensitivity to sample size, it can be used as an index of goodness of fit (with recommended values ranging between 2 and 5). Also, RMSEA values of less than 0.05 are indicative of a close fit, and values lower than 0.11 indicate an acceptable fit. As for the GFI and AGFI, values of 0.90 or greater indicate well-fitted models [65]. The confirmatory factor analysis was done using the STATISTICA software version 12.

Cronbach’s alpha test assessed the reliability of the suicidal ideation score. The cut-offs for reliability were as follow: poor (less than 0.6), moderate (between 0.6 and 0.7), good (between 0.7 and 0.8), very good (between 0.8 to 0.9), excellent (higher than 0.9) [66]. A Spearman correlation between C-SSRS and the other measures was done to establish convergent validity. The Correlation coefficient values of │0.1–0.3│, │0.3–0.7│, and > │0.7│ indicate weak, moderate, and strong correlations, respectively [67]. Linear regression was performed, having the dependent variable as the suicidal ideation score. All variables were included in the multivariable model.

Furthermore, since the suicidal ideation score was not normally distributed (as verified by the Shapiro Wilk test), non-parametric tests were used (Kruskal-Wallis for comparing three groups or more and Mann-Whitney tests for comparing two groups). Multiple linear regression was performed using the suicidal ideation score as the dependent variable. All variables were included in the multivariable model. In all cases, a value of p < (0.05) was considered statistically significant.

## Results

Table 1 presents the sociodemographic features of the participants. The mean age was 30.30 ± 12.52 years, with 54.8% males. The majority (70.9%) had a university level of education, 63.1% were single, and 50.7% earned less than 1000 US dollars per month.

**Table 1:** Sociodemographic characteristics of the sample population

	Frequency (%)
Gender	
Male	423 (54.8%)
Female	349 (45.2%)
Education level	
Illiterate (no schooling/less than primary education)	12 (1.6%)
Primary	39 (5.3%)
Complementary	52 (7.0%)
Secondary	113 (15.2%)
University	526 (70.9%)
Socioeconomic status	
< 1000 $	376 (50.7%)
1000–2000 $	260 (35.1%)
> 2000 $	105 (14.2%)
Marital status	
Single	488 (63.1%)
Married	236 (30.5%)
Widowed	19 (2.5%)
Divorced	30 (3.9%)
	**Mean ± SD**
Age (in years)	30.30 ± 12.52

### Description of the used scales

Of the total sample, 228 (28.9%) participants had suicidal ideations, with a mean suicidal ideation score of 0.57 ± 1.16, 19.8% of participants wished to be dead, 9.6% had suicidal thoughts, 13.8% suicidal thoughts with a method, 25.5% suicidal intent, and 23.3% suicidal intent with a specific plan (Table 2). The mean, SD, and the range of the scales used are presented in Table 3.

**Table 2:** Description of the suicidal ideation scale

	Frequency (%)
Suicide ideation (Most severe in the past week)	
1. Wish to be dead	
Yes	144 (19.8%)
No	582 (80.2%)
2. Suicidal thoughts	
Yes	71 (9.6%)
No	671 (90.4%)
3. Suicidal thoughts with a method (but without specific plan or intent to act)	
Yes	66 (13.8%)
No	414 (86.2%)
4. Suicidal intent (without specific plan)	
Yes	82 (25.5%)
No	239 (74.5%)
5. Suicidal intent with a specific plan	
Yes	66 (23.3%)
No	217 (76.7%)
**Suicidal ideation score**	
Presence of suicidal ideation	228 (28.9%)
Absence of suicidal ideation is present	561 (74.8%)

**Table 3:** Description of the used scales

	Mean	SD	Median	Interquartile range (IQR)	Minimum	Maximum	Range
Suicidal ideation score	0.57	1.16	0.001	1.00	0	5	0–5
Audit score	10.36	8.21	8.00	14.00	0	39	0–40
TAS score	52.11	10.43	52.00	14.00	20	91	20–100
HAM-D score	12.67	10.50	11.00	16.00	0	44	0–52
Self-esteem score	24.67	4.63	25.00	3.00	10	37	10–40
HAM-A score	14.19	10.10	15.00	16.50	0	47	0–56
PSC score	18.50	5.92	19.00	7.00	0	37	0–40
LSAS	39.71	2.91	37.00	30.00	0	128	0–144
EI	81.93	29.35	81.00	37.00	0	160	0–160

*TAS* Toronto Alexithymia Scale, *HAMD* Hamilton depression rating scale (HDRS), *HAMA* Hamilton anxiety scale, *PSC* Perceived Stress Scale, *LSAS* Liebowitz Social Anxiety Scale, *EI* Emotional intelligence total score

### Construct validity

A factor analysis using the promax rotated matrix for the C-SSRS was run over the whole sample. The results showed that all variables could be extracted from the questions asked. The C-SSRS scale converged over a solution of one factor (Eigenvalue >| 1), the proportion of explained variance was 0.797 (KMO = 0.899; Bartlett’s test of sphericity *p* < 0.001) (Table 4).

**Table 4:** Factor analysis of the Columbia – suicide severity rating scale items using the promax rotation

		Factor 1	Communality
Item 1	Wish to be Dead	0.867	0.751
Item 2	Non-Specific Active Suicidal Thoughts	0.921	0.848
Item 3	Active Suicidal Ideation with Any Methods (Not Plan) without Intent to Act	0.900	0.811
Item 4	Active Suicidal Ideation with Some Intent to Act, without Specific Plan	0.919	0.844
Item 5	Active Suicidal Ideation with Specific Plan and Intent	0.854	0.730
**Cronbach’s alpha**		0.796	
**Proportion of variance explained**		0.797	

The confirmatory factor analysis showed that the Maximum Likelihood Chi-Square = 7.55 and Degrees of Freedom = 5, resulting in a χ2/df = 1.51. The Steiger-Lind RMSEA was equal to 0.052, while the Joreskog GFI and AGFI were 0.986 and 0.957, respectively.

### Convergent validity

The convergent validity with the C-SSRS was tested with the mental scales used. The results showed a moderate positive correlation between the suicide ideation score and the HDRS (rho = 0.507, *p* < 0.001) and the HAM-A (rho = 0.402, p < 0.001). Moreover, a moderate positive correlation was found between suicide ideation score and alcohol dependence (rho = 0.393, *p* < 0.001), while a moderate negative association was found between suicide ideation score and emotional intelligence (rho = − 0.318, *p* < 0.001). A weak positive correlation was found between suicide ideation score and alexithymia (rho = 0.137, *p* < 0.001), perceived stress (rho = 0.143, p < 0.001), and social anxiety (rho = 0.121, *p* = 0.002). However, a weak negative correlation was found between suicide ideation score and self-esteem score (rho = − 0.271, *p* < 0.001) (Table 5).

**Table 5:** Correlation matrix among the scales used

	Suicide ideation score	Audit score	TAS score	HAM-D score	Self-esteem score	HAM-A score	PSC score	LSAS	EI
Suicide ideation score	–	0.393*	0.137*	0.507*	−0.271*	0.402*	0.143*	0.121*	−0.318*
Audit score	0.393*	**–**	0.274*	0.509*	− 0.249*	0.411*	0.165*	0.165*	− 0.312*
TAS score	0.137*	0.274*	**–**	0.284*	−0.307*	0.349*	0.388*	0.138*	−0.122*
HAM-D score	0.507*	0.509*	0.284*	**–**	−0.385*	0.668*	0.250*	0.297*	−0.394*
Self-esteem score	−0.271*	−0.249*	− 0.307*	−0.385*	–	− 0.375*	−0.240*	− 0.262*	0.426*
HAM-A score	0.402*	0.411*	0.349*	0.668*	−0.375*	**–**	0.342*	0.277*	−0.346*
PSC score	0.143*	0.165*	0.388*	0.250*	−0.240*	0.342*	**–**	0.109*	−0.135*
LSAS	0.121*	0.165*	0.138*	0.297*	−0.262*	0.277*	0.109*	**–**	−0.248*
EI	−0.318*	−0.312*	0.122*	−0.394*	0.426*	−0.346*	− 0.135*	−0.248*	**–**

*TAS* Toronto Alexithymia Scale, *HAMD* Hamilton depression rating scale (HDRS), *HAMA* Hamilton anxiety scale, *PSC* Perceived Stress Scale, *LSAS* Liebowitz Social Anxiety Scale, *EI* Emotional Intelligence total score

Values marked in bold are significant. * *p* – value less than 0.01

### Bivariate analysis

The results of the bivariate analysis are summarized in Table 6. The mean of the C-SSRS scale was higher in male participants than in females (0.69 vs. 0.40, *p* < 0.001). It was also higher among widowed than single participants (1.78 vs. 0.50, p < 0.001). No significant correlation was found between suicide ideation score and age (rho = − 0.05, *p* = 0.885).

**Table 6:** Bivariate analysis of the factors associated with the C-SSRS scale

	**C-SSRS scale**	***p*-value**
	**Mean ± SD**	
Gender		
Male	0.69 ± 1.33	**< 0.001**
Female	0.40 ± 0.88	
Marital status		
Single	0.50 ± 1.12	**< 0.001**
Married	0.51 ± 1.04	
Widowed	1.78 ± 1.61	
Divorced	0.72 ± 1.30	
Socioeconomic status		
< 1000 $	0.50 ± 1.12	0.171
1000–2000 $	0.58 ± 1.19	
> 2000 $	0.75 ± 1.23	
Education level		
Illiterate	0.75 ± 1.54	0.707
Primary	0.71 ± 1.27	
Complementary	0.62 ± 1.15	
Secondary	0.58 ± 1.27	
University	0.50 ± 1.09	
	**Correlation coefficient**	***p*-value**
Age	−0.05	0.885

Numbers in bold indicate significant *p*-values

### Multivariable analysis

A linear regression taking the C-SSRS scale as the dependent variable showed that higher depression (Unstandardized Beta, B = 0.035, p < 0.001), higher anxiety (B = 0.015, *p* = 0.008), and higher alcohol dependence (B = 0.024, p < 0.001) were significantly linked to higher suicidal ideation score. However, higher self-esteem (B = -0.041, *p* = 0.006) was significantly associated with lower suicidal ideation scores (Table 7).

**Table 7:** Multivariable analysis

Model 1: Linear regression taking the C-SSRS scale as dependent variable and the scales as independent variables.					
	Unstandardized Beta	Standardized Beta	***p***-value	Confidence interval	
				Lower Bound	Upper Bound
Gender (Male* vs Female)	−0.115	−0.051	0.170	−0.278	0.049
Marital status (Single* vs Married)	−0.017	−0.007	0.875	−0.229	0.195
Intermediate monthly income	0.058	0.024	0.535	−0.126	0.243
High monthly income	0.044	0.013	0.740	−0.215	0.302
Age	−0.007	−0.074	0.103	−0.015	0.001
**AUDIT score**	**0.024**	**0.177**	**< 0.001**	**0.013**	**0.036**
Alexithymia	−0.005	−0.051	0.218	−0.014	0.003
**Self esteem**	**−0.041**	**−0.098**	**0.006**	**−0.070**	**− 0.012**
**Depression score**	**0.035**	**0.312**	**< 0.001**	**0.024**	**0.046**
**Anxiety score**	**0.015**	**0.130**	**0.008**	**0.004**	**0.026**
Self-esteem score	0.002	0.014	0.740	−0.012	0.017
Social anxiety score	−0.002	− 0.031	0.413	− 0.005	0.002
Emotional intelligence score	−0.002	−0.053	0.167	−0.005	0.001

Variables entered: Gender, marital status, socioeconomic status, age, alcohol dependence, alexithymia, depression and anxiety scales, perceived stress scale, self-esteem scale, social anxiety scale and emotional intelligence

Numbers in bold indicate significant *p*-values

## Discussion

The current study determined the psychometric characteristics of the C-SSRS translated into Arabic and examined the risk factors associated with suicide ideation among Lebanese adults. This scale exhibited adequate construct validity and internal consistency. The results also showed that depression, anxiety, and alcohol dependence were positively associated with suicidal ideation.

### Validation of the C-SSRS

The results showed that the Arabic version of the C-SSRS has satisfactory psychometric properties with adequate internal consistency, which offers initial evidence that it could be used in Lebanon to screen for suicidal ideation. The construct validity of the C-SSRS scale was adequate since items converged over one factor. The validated Spanish version of the C-SSRS had an adequate internal consistency of 0.87, and the factor loading of the scale yielded two items factors for suicidal thoughts and behavior, respectively [68]. The first factor included ten items, consistent with the concept of suicide ideation [68]. The concurrent validity of the Spanish scale has found a positive relationship with depression [68], similar to our results. The Cronbach’s alpha value for the C-SSRS scale was good equal to 0.797, which is consistent with a study that found Cronbach’s alpha values ranging between 0.73 and 0.93, indicating good internal consistency for the suicide ideation subscale of the C-SSRS [48]. The wide variations across studies are due to differences in the characteristics of the sample, the population surveyed, and the scale used. Our findings revealed that the means of items of the suicidal ideation subscale ranged from 0.09 to 0.25, with the highest reported frequency for item 4 (suicidal intent without a specific plan), consistent with the results of the Spanish study, showing means ranging from 0.11 to 0.15 [68].

Our results revealed a moderate convergent validity between the suicidal ideation score and depression and anxiety. A previous study had demonstrated a good convergent validity between the C-SSRS and items on the Beck Depression Inventory (BDI) and the Montgomery-Åsberg Depression Rating Scale (MADRS) [48]; this discrepancy in results could be due to the difference in the scales used to assess depression and anxiety. Another explanation would be that both the BDI and MADRS include questions about suicidal ideation, leading to a better correlation with the C-SSRS. Moreover, depression and anxiety were related to suicidal ideation score, consistent with previous findings showing that both are correlated with suicidal ideation and increased thoughts of suicide [69–73].

### Suicide ideation correlates

Our results showed that the prevalence of suicidal ideation among Lebanese adults was 28.9%, in line with those of a previous study among Lebanese adolescents [41] but higher than the findings of other studies [74, 75]. Indeed, the overall prevalence of suicidal ideation in 12 Muslim-majority countries was 22%, with 12% in the 87 Lebanese participants [42]. It is noteworthy that all religions prohibit suicide and that the majority of people in Middle Eastern countries are practicing believers, which makes suicide a taboo in this part of the world, leading to a failure in collecting data about suicide and decreasing the prevalence of suicidal ideation and attempts [42, 74, 75]. The low number of participants in this study can explain the discrepancy with our results. However, further studies are needed to assess the reasons for these differences, including the nature of the sample.

Our results also showed that depression is highly correlated to suicidal ideation compared to the other variables examined, followed by anxiety. These findings are consistent with those of a previous study conducted among Tehran university students, showing that depression was the first contributor to suicidal ideation followed by anxiety; mental health, resiliency, and daily stress were respectively the other contributors [76]. Depressive symptoms are predictors of suicidal ideation in 94% of cases [76, 77]. Suicide is strongly related to mental disorders, such as depression, and other factors like anxiety are contributors [5]. Most of the people who committed suicide had mental disorders, among which depression [78]. This strong relationship between suicide and depression is not yet explored, and more studies are needed to clarify the characteristics of depression and its effects on suicide.

Our results showed that higher alcohol dependence was significantly associated with more suicidal ideation, in agreement with those of previous studies showing that alcohol abuse increases suicidal ideation, causes psychiatric diseases, and negatively affects mental health, particularly depression [29, 79, 80]. Previous findings from Lebanon had demonstrated the association between alcohol dependence and mental health, including depression [81], alexithymia [70, 82] and stressful living situations [83], but not suicidal ideation.

### Clinical implications

Our findings support the need for clinical interventions to decrease the psychological distress linked to suicidal ideation. Additionally, the intervention on emotional intelligence might be useful to improve mental health and reduce psychological distress factors. Emotional intelligence seems to be a preventive factor that contributes to breaking the negative emotions of the person, such as depression, anxiety, and other suicide-related factors [84–86].

### Limitations

Although our results are in agreement with those of previous papers, our study has some limitations. It has a cross-sectional methodology and uses self-report questionnaires as the only form of assessment, leading to a risk of reverse causality and information bias, in addition to the lack of temporality. Moreover, it did not apply external measures of suicidal ideation, behavior, or risk to test for convergent validity. Also, the sample may not be representative of the general population, as the majority of the participants were young adults, single, with a university level of education. The prevalence of suicidal ideation was relatively high and could be a biased estimate of suicidal ideation in the target population due to the lack of representativeness. Furthermore, the response to suicidal ideation questions was either yes or no, and did not assess its severity; in depth-interviews inquiring about the reasons for suicidal ideation are needed for a better evaluation. Further studies considering all these limitations are warranted to confirm our findings.

## Conclusion

Our findings suggest that the Arabic version of the C-SSRS subscale could serve as an appropriate assessment tool for suicidal ideation. This paper also gave insights into factors correlated with higher suicidal ideation scores, such as depression, anxiety, and alcohol dependence. Further studies are necessary to confirm our findings and implement suicide prevention programs.

## Data Availability

The authors do not have the right to share any data information as per the ethics committee rules and regulations.

## Abbreviations

AUDIT: Alcohol Use Disorders Identification Test
C-SSRS: Columbia-Suicide Severity Rating Scale
HAM-A: Hamilton anxiety scale
HDRS: Hamilton depression rating scale
KMO: Kaiser–Meyer–Olkin
LSAS: Liebowitz Social Anxiety Scale
PSS: Perceived Stress Scale
RSES: Rosenberg self-esteem scale
TAS-20: Toronto Alexithymia Scale
